# Downregulation of SPARC Expression Inhibits the Invasion of Human Trophoblast Cells In Vitro

**DOI:** 10.1371/journal.pone.0069079

**Published:** 2013-07-23

**Authors:** Yahong Jiang, Yan Zhu, Yan Shi, Yaping He, Zhichao Kuang, Zhaogui Sun, Jian Wang

**Affiliations:** NPFPC Key Laboratory of Contraceptives and Devices, Shanghai Institute of Planned Parenthood Research, Shanghai, China; State Key Laboratory of Reproductive Biology, Institute of Zoology, Chinese Academy of Sciences, China

## Abstract

Successful pregnancy depends on the precise regulation of extravilloustrophoblast (EVT) invasion into the uterine decidua. SPARC (secreted protein acidic and rich in cysteine) is a matricellular glycoprotein that plays critical roles in the pathologies associated with obesity and diabetes, as well as tumorigenesis. The objective of this study was to investigate the role of SPARC in the process of trophoblast invasion which shares many similarities with tumor cell invasion. By Western blot, higher expression of SPARC was observed in mouse brain, ovary and uterus compared to other mouse tissues. Immunohistochemistry analysis revealed a spatio-temporal expression of SPARC in mouse uterus in the periimplantation period. At the implantation site of d8 pregnancy, SPARC mainly accumulated in the secondary decidua zone (SDZ), trophoblast cells and blastocyst. The expression of SPARC was also detected in human placental villi and trophoblast cell lines. In a Matrigel invasion assay, we found SPARC-specific RNA interference significantly reduced the invasion of human extravilloustrophoblast HTR8/SVneo cells. Microarray analysis revealed that SPARC depletion upregulated the expression of interleukin 11 (IL11), KISS1, insulin-like growth factor binding protein 4 (IGFBP4), collagen type I alpha 1 (COLIA1), matrix metallopeptidase 9 (MMP9), and downregulated the expression of the alpha polypeptide of chorionic gonadotropin (CGA), MMP1, gap junction protein alpha 1 (GJA1), et al. The gene array result was further validated by qRT-PCR and Western blot. The present data indicate that SPARC may play an important role in the regulation of normal placentation by promoting the invasion of trophoblast cells into the uterine decidua.

## Introduction

Embryo implantation, a complex physiological process, depends on a series of key events, including blastocyst apposition and adhesion to the luminal epithelium, extensive degradation and remodeling of extracellular matrix, invasion of the trophoblast cells into the maternal endometrium, and secretion of local cytokines to activate dialogue between the maternal endometrium and the implanting blastocyst [1], [2]. Because of ethical restrictions and limited availability of human placental tissue, our understanding of implantation comes mostly from *in vitro* experiments using cultured human trophoblasts or cell lines, mainly derived from choriocarcinoma. It has been proposed that embryonic extravilloustrophoblast (EVT) are similar to cancer cells [3]. However, in contrast to cancer cells, EVT invasion during normal pregnancy is precisely regulated both spatially and temporally [4]. Such a precise invasion involves complex and synchronized molecular and cellular events between uterus and implanting embryo [5], [6], which are regulated by paracrine and autocrine factors. It has also been postulated that cell adhesion molecules, extracellular matrix (ECM) proteins, growth factors, cytokines, hormones, inflammatory factors, and extracellular degrading matrix proteinases are involved in embryo implantation [7].

SPARC (secreted protein acidic and rich in cysteine), also known as osteonectin and BM-40, is a matricellular glycoprotein that modulates ECM assembly and turnover in many physiological processes [8], [9]. SPARC interacts with several extracellular matrix components and functions as a de-adhesive molecule, a cell cycle inhibitor and a modulator of cytokine and growth factor activities. SPARC is spatially and temporally regulated during development and expressed at high levels in remodeling tissues [10], [11]. It is a key player in the pathologies associated with obesity and diabetes [12]. In addition, SPARC modulates angiogenesis through interfering with the binding of angiogenic stimulators, vascular endothelial growth factor (VEGF), platelet-derived growth factor (PDGF), and basic fibroblast growth factor (bFGF) to their receptors in endothelial cells [13]. The role of SPARC in tumorigenesis appears to be cell-type specific due to its diverse function in a given microenvironment [14]. In melanoma cells, high level of SPARC expression induces epithelial-mesenchymal transition and increases invasion and tumor progression [15], [16]. High levels of SPARC are also associated with invasive meningioma, osteosarcoma and glioma [17], [18], [19], [20]. On the other hand, in neuroblastoma and breast, pancreatic, lung and ovarian cancers, SPARC functions as a tumor suppressor [21].

We found that the expression of SPARC at the implantation sites was up-regulated compared to the inter-implantation sites in uteri from day 8 pregnant mice according to tissue microarray analysis (unpublished data). Based on the critical role of SPARC in tumor invasion and progression, we hypothesized that SPARC may also play a role in regulation of blastocyst implantation, especially in the process of trophoblast invasion which shares many similarities with invasion of tumor cells. Thus, we examined the expression patterns of SPARC in mouse uterus during early pregnancy as well as in two established cell models of trophoblast cell lineage. The effects of SPARC on the invasion of human trophoblast cells were also determined by RNA interference.

## Materials and Methods

### Ethics Statement

All the experiments were carried out in accordance with the Guidelines for the Care and Use of Laboratory Animals, and were approved by the Ethics Committee of Shanghai Institute of Planned Parenthood Research (SIPPR). Placental tissues were collected in the Special Hospital of SIPPR, and the procedures were in accordance with guidelines established by the Ethics Committee of SIPPR. All participants provided written informed consents. Ethical approval was granted by the ethical committee of the SIPPR (2010–11).

ICR female and male adult mice (56 days postnatal) were obtained from the Sino-British Sippr/BK Lab Animal Ltd, Shanghai, China. All the mice were raised in a controlled environment, with a temperature range between 22 to 24°C and a 14 h light and 10 h dark photoperiod. The mice were mated to induce pregnancy (the day when the vaginal plug was visualized was designated as day 1 of pregnancy).

### Cell culture and RNA interference

HTR8/SVneo trophoblast line was developed from an explant culture of human first-trimester placenta and were immortalized by introducing the gene encoding simian virus 40 large T antigen [22]. The cells were maintained in RPMI1640 medium containing 10% fetal bovine serum (FBS), 1 mmol/l sodium pyruvate, 2 mmol/l L-glutamine, 100 μg/ml streptomycin, and 100 U/ml penicillin. The choriocarcinoma cell line JEG-3 was maintained in F-12/Dulbecco's modified Eagle's medium (1∶1) containing 10% fetal bovine serum, 100 U/ml penicillin, and 100 μg/ml streptomycin. All cells were maintained at 37°C with 5% CO_2_.

For RNA interference, the 25-nucleotide synthetic duplexes of SPARC siRNA were prepared by Invitrogen (Gaithersburg, MD, USA). Sequences of SPARC specific siRNA are: 1, AGUCACCUCUGCCACAGUUUCUUCC; 2, AUACAGGGUGACCAGGACGUUCUUG; 3, AUUCUCAUGGAUCUUCUUCACCCGC. HTR8/SVneo cells were transfected with SPARC siRNA or a 25-nucleotide universal negative control siRNA using Lipofectamine 2000 (Invitrogen) according to the manufacturer's protocol. Twenty-four hours after transfection, cells were subject to migration and invasion assays.

### RNA extraction, RT-PCR and qRT-PCR

Total cellular RNA from 2.0×106 cells was extracted using Trizol reagent (Invitrogen) according to the manufacturer's instructions. The first strand cDNA was synthesized with Superscript II reverse transcriptase (Invitrogen) and oligodT from 2 µg of total RNA. All PCR reactions were conducted in the exponential range of amplification for each set of primers. Specific PCR primer pairs and the different parameters of amplification are summarized in Table 1. RT-PCR reactions were performed in a final volume of 25 µl containing 2 µl of the first strand cDNA, 200 µmol/l dNTPs, 2 mmol/l MgCl2, 1 U Taq polymerase (TaKaRa Corp., Dalian, China), and 10 pmol of each primer. As negative controls for PCR, samples without first strand cDNA or without Taq enzyme were used. Amplification of GAPDH gene transcripts was used to confirm RNA integrity and efficiency. All control reactions yielded negative results.

**Table 1:** The sequences of specific PCR primer pairs for PCR amplification.

Gene	Primer sequence (5′→ 3′)	Product (bp)
SPARC	F-GTA CAT CGC CCT GGA TGA GT	178
	R-TGT CTC CAG GCA GAA CAA CA	
COLIA1	F-GGC CCA GAA GAA CTG GTA CA	210
	R-ATG TAG GCC ACG CTG TTC TT	
IL11	F-CTG TGG GGA CAT GAA CTG TG	212
	R-CGT CAG CTG GGA ATT TGT C	
IGFBP4	F-CCC ACG AGG ACC TCT ACA TC	172
	G-CAG TCC AGC TCC CCC TTT	
MMP9	F-TGA CAG CGA CAA GAA GTG GG	208
	R-TTC AGG GCG AGG ACC ATA GA	
MMP1	F-ACA CGC CAG ATT TGC CAA GAG CA	463
	R-GGG GTT TGT GGG CCG ATG GG	
TIMP3	F-CTG ACA GGT CGC GTC TAT GA	240
	R-GGC GTA GTG TTT GGA CTG GT	
KISS1	F-CCC AGG CCA GGA CTG AGG CA	173
	R-AGC TGC TGG CCT GTG GGT CT	
GJA1	F-TCA TTA GGG GGA AGG CGT GAG GA	286
	R-TCC CCA GCA GCA GGA TTC GGA	
TGFBR3	F-CTG CCT GCC CTG CAG AAC CC	391
	R-CTG ACC ACC GGG GCC GAG TA	
GAPDH	F-AGC CAC ATC GCT CAG ACA C	315
	R-TGG ACT CCA CGA CGT ACT C	

Quantitative real-time PCR (qRT-PCR) was performed with SYBR Green Real time PCR Master Mix (QPK-201, Toyobo Co., Ltd., Osaka, Japan) using a Bio-Rad Chromo4 real-time PCR system (Bio-Rad, California, USA). Samples were run in triplicate to ensure amplification integrity. The standard PCR conditions were as follows: 95°C for 60 s, then 40 cycles at 95°C for 15 s, 58°C for 15 s and 72°C for 45 s. The threshold for positive of real-time PCR was determined based on negative controls. The expression levels of genes were normalized to the expression level of GAPDH mRNA in each sample and evaluated by 2−ΔΔCT method (Livak and Schmittgen, 2001).

### Western blot analysis

Whole protein extracts were prepared in lysate buffer (50 mmol/l HEPES, 150 mmol/l NaCl, 1 mmol/l EGTA, 1.5 mmol/l MgCl2, 100 mmol/l NaF, 10% glycerol and 1% Triton X-100, 1 mmol/l PMSF, 10 µg/ml aprotinin and 1 mmol/l sodium orthovanadate). Protein concentration was determined by DU530 UV spectrophotometer (Beckman, Fullerton State, CA, USA). Equal amounts of protein (50 µg) from different treatments were boiled in 20 µl loading buffer (100 mmol/l Tris, pH 6.8, 4% SDS, 20% glycerol, 10% beta-mercaptoethanol, 0.2% bromophenol blue), fractionated by electrophoresis in 12% SDS polyacrylamide gels under reducing conditions, and transferred to nitrocellulose membranes. After blocking in 5% BSA solution, the membrane was immunoblotted with antibodies against SPARC (Santa Cruz, sc25574) at 1∶1000, anti-TIMP3 at 1∶1000 (Abcam, ab39184), anti-MMP9 at 1∶3000 (Abcam, ab38898), anti-IL11 at 1∶500 (Abcam, ab76589), anti-MMP1 at 1∶200 (Abcam, ab38929) , anti-MMP2 at 1∶200 (Abcam, ab7032) and anti-GAPDH (Abcam, ab37187) at 1∶5000, and HRP-conjugated goat anti-rabbit or anti-mouse secondary antibody (Santa Cruz, sc2004, sc2005) at 1∶5000. Color development was performed using the Enhanced Chemiluminescence System (Pierce, Rockford, IL, USA).

### Histological analysis and Immunohistochemistry

Tissue specimens were fixed in freshly made 4% buffered paraformaldehyde in PBS at 4°C for more than 40 h, which were then dehydrated in graded alcohol and embedded in paraffin (Beijing Chemical, Beijing, China). Sections of specimens were processed for immunohistochemical detection with a Histostain-Plus Kit and diaminobenzidine (DAB; Zhongshan Corp., Beijing, China) according to the manufacturer's protocol. Briefly, sections (5 µm) were deparaffinized and rehydrated in xylene and a graded series of ethyl alcohol, and rinsed in PBS. Antigen retrieval was performed by placing the slides in boiling citric acid buffer (10 mmol/l of citrate sodium and 10 mmol/l of citric acid) for 15 min. The sections were cooled to room temperature and sequentially incubated at room with 3% H2O2 in methanol for 15 min to quench endogenous peroxidase and in normal blocking serum for 30 min. The sections were then incubated with rabbit anti-SPARC (1∶200) primary antibody overnight at 4°C, stained with DAB, and finally counterstained with hematoxylin-eosin (Sigma). Intervening PBS washes were performed after incubation when necessary. Biotin-SP-conjugated donkey anti-rabbit IgG antibody (1∶200 in blocking solution, Proteintech Company) was used as the secondary antibody. For negative controls, 10% donkey serum was used instead of primary antibodies. The degree of staining was subjectively assessed by blind examination of the slides by three investigators independently. All the sections were examined and photographed under the microscope (DFC420C, Leica, Germany).

### Matrigel cell invasion and migration assay

Matrigel invasion assay was prepared as previously described in detail [23], [24]. Cells were transfected with control (CTL) siRNA or SPARC siRNA for 24 h at 37°C before being performed for invasion assay. Invasion of HTR8/SVneo cells was measured in Matrigel (BD Biosciences, Beit-Ha' Emek, Israel) coated transwell inserts (6.5 mm filters; Costar, Cambridge, MA, USA) containing polycarbonate filters with 8 μm pores. The transwell inserts were coated with 50 µl of 1 mg/ml Matrigel matrix according to the manufacturer's recommendations. After incubation at 37°C, 4 h for gelling, 105 cells in 200 µl RPMI1640 medium without fetal bovine serum were plated in the upper chamber on top of the Matrigel, whereas 600 µl of medium with 10% fetal bovine serum were added to the lower well. After incubation for 24 h, the cells on the upper well were completely removed by cotton swab, and the invaded cells attached to the bottom side of the filter were fixed with methanol and stained with hematoxylin and eosin. Cell invasion ability was determined by counting the number of stained cells attached on the other side of the filter in ten randomly selected non-overlapping fields on the membranes at a magnification of x200. Invasion of cells under different treatments was normalized to the control and expressed as the mean invasion (% invasion ± SEM). The migration assay was identical to the invasion assay mentioned above except that the transwell chambers were not coated with Matrigel.

### MTT assay

HTR8/SVneo cells were transfected with CTL siRNA or SPARC siRNA using Lipofectamine 2000. Forty eight hours after transfection, cells (1×10^4^) were harvested and plated in 96-well plate for 20 h before incubation with MTT solution (ZhongshanCorp, Beijing, China) for an additional 4 h. DMSO was then added to the culture wells to solubilize the reactive crystals, and the absorbance at 595 nm was recorded using a 96-well plate reader (Bio-Tek, Vermont, USA).

### cDNA microarray analysis

RNA was isolated from fresh frozen HTR8/SVneo cells using standard QIAGEN Rneasy procedures (Qiagen Inc, Valencia, CA). For microarray analysis, 10 µg of total RNA was used in a reverse transcription reaction to generate first-strand cDNA using the SuperScript choice system (Invitrogen). *In vitro* transcription reaction of cDNA to cRNA was performed overnight (14 h) including biotin-11-dUTP for labeling of the cRNA product. Hybridization to human WG-6v3 Expression BeadChip (Illumina, San Diego, CA) was conducted at Biostar Genechip Inc. (Shanghai, China) using standard Illumina protocols. In brief, 750 ng of labeled cRNAs were hybridized to the Human WG-6 v3 Beadchip arrays at 55°C overnight (∼16 h) following the Whole-Genome Gene Expression Protocol for BeadStation (Illumina) and stained with 1 μg/ml streptavidin-Cy3 (Amersham Biosciences, Piscataway, NJ) for visualization. The human WG-6 v3 BeadChips contain sequences representing >27,000 curated genes. Quality standards for hybridization, labeling, staining, background signal, and basal level of housekeeping gene expression for each chip were verified. After scanning the probe array, the BeadScan image data were acquired and analyzed using the BeadStudio software (Illumina). The data discussed in this publication have been deposited in National Center for Biotechnology Information's Gene Expression Omnibus (GEO) and are accessible through GEO Series accession number GSE37639.

### Statistical analysis

The Student *t*-test was used to evaluate the difference between groups, and *p*-values of <0.05 (2-sided) were considered significant. Results are expressed as means ± SD.

## Results

### SPARC expression pattern in the mouse uterus in the periimplantation period

**Figure 1:**
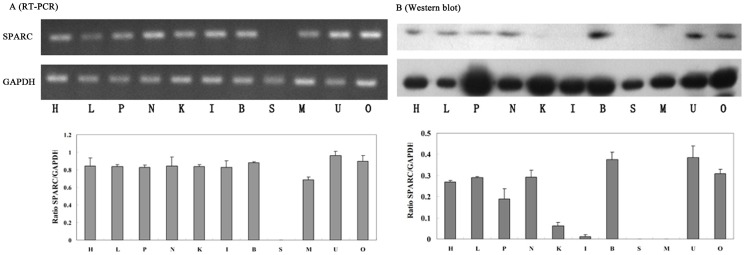
Tissue distribution analysis of SPARC in ICR mice. Samples were isolated from heart (H), liver (L), spleen (P), lung (N), kidney (K), intestinal (I), brain (B), skin (S), muscle (M), uteri (U) and ovary (O). GAPDH was an internal control for RT-PCR and loading control for Western blot (here and after). *Columns*, mean of triplicates; *bars*, SD. Representative of three independent experiments.

In order to determine a possible role of SPARC in pregnancy, we first examined the tissue specificity of SPARC by RT-PCR and Western blot. As shown in Fig. 1, SPARC is widely expressed in mouse tissues. Uterus, ovary and brain showed the highest levels of both RNA (A) and protein (B) compared to the heart, liver, spleen and lung, which had moderate amounts of SPARC whereas kidney, intestinal, muscle and skin tissue showed minimal expression. The high level of SPARC expression in the uteri and ovary indicates a potential role in reproduction.

**Figure 2:**
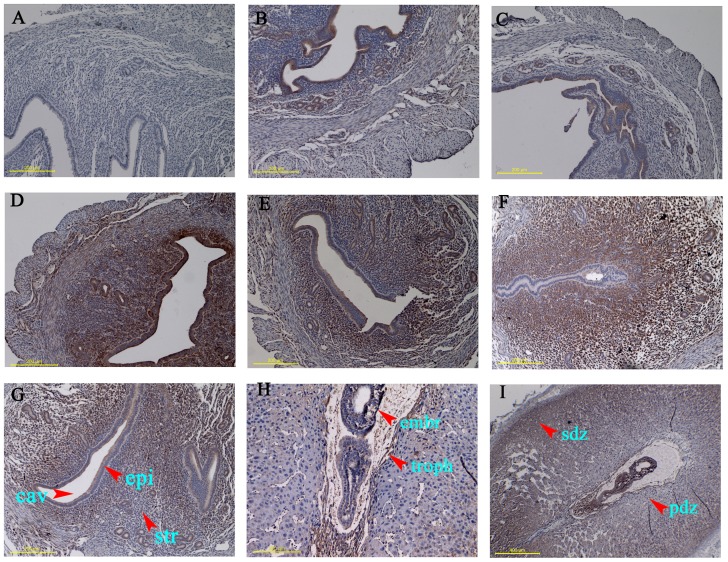
Immunohistochemical study showing the distribution of SPARC protein in mouse uteri during the early pregnancy. Mouse uteri were collected on day 0 (B), 1 (C), 4 (D), 5 (E,F) and 8 (G,H) of pregnancy. A: negative control with normal serum was used in place of anti-SPARC antibody; E,G: inter-implantation sites; F,H: implantation sites; I is a shrunken picture of H. *cav*, uterine cavity; *epi*, endometrial epithelia; *str*, endometrial stroma; *embr*, embryo; *troph*, trophoblast; *pdz*: primary decidua zone; *sdz*: secondary decidua zone. Representative of three independent experiments.

Expression of SPARC in uterus of mouse during periimplantation period was examined to investigate if changes in expression correlate with ongoing implantation process. Immunohistochemistry analysis was performed to assay uterine samples of mice from day 0 to day 8 of pregnancy (Fig. 2). Negative control of IHC staining did not show any staining (Fig. 2A). On day 0 and day 1 of pregnancy, distinct signals were evident primarily in the luminal and glandular epithelia (Fig. 2, B and C), On day 4, accumulation of this protein was noted in the stromal cells in addition to its localization in epithelial cells, and the signals were more intense (Fig. 2D). The patterns of SPARC accumulation at inter-implantation sites of day 5 (Fig. 2E) and day 8 (Fig. 2G) were similar to that of day 4 except that the signals slightly decreased. We found at day 5 (Fig. 2F) SPARC mainly accumulated in primary decidua zone (PDZ) and stromal cells, while at day 8 mainly accumulated in the secondary decidua zone (SDZ), trophoblast cells and blastocyst at the implantation sites (Fig. 2, H and I).

### Expression of SPARC in human placental villi and in trophoblast cells

**Figure 3:**
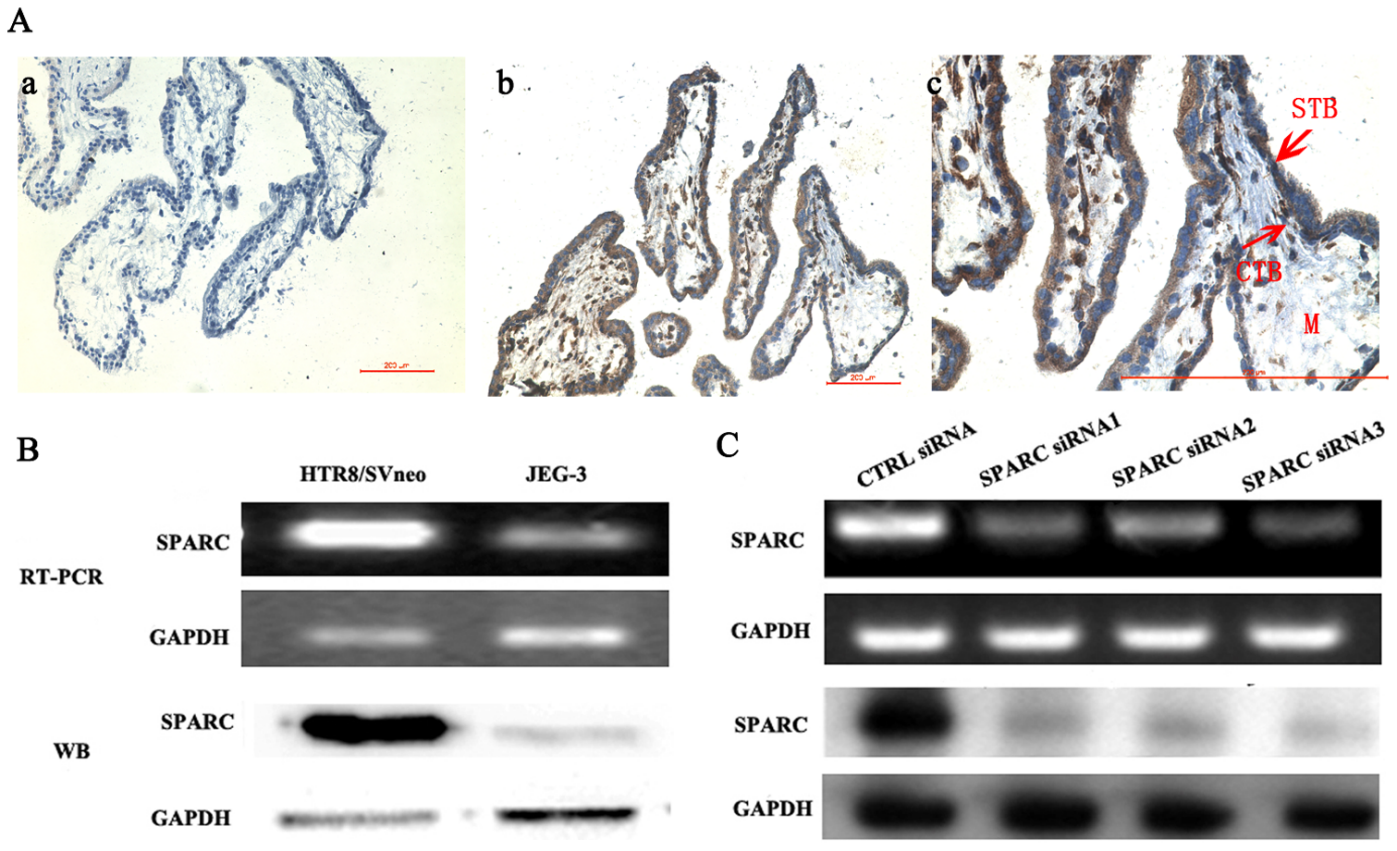
SPARC siRNA inhibited the expression of SPARC in HTR8/Svneo cells. A. Expression of SPARC in human placental villa at 8 weeks of gestation. (a) negative control with normal serum was used in place of anti-SPARC antibody; (b) placental villa immunostained with anti-SPARC antibody; (c) is an amplified picture of (b). Bar represents 200 µm. *ctb*, cytotrophoblast cell; *stb*, syncytiotrophoblast cell; *m*, villous mesenchymal cell. B. Expression of SPARC in human trophoblast cell lines HTR8/SVneo and JEG-3. C. Transfection HTR8/SVneo cells with SPARC siRNA inhibited the expression of SPARC at both mRNA level and protein level. Representative of three independent experiments.

To investigate the possible roles of SPARC in regulating human blastocyst implantation, we first examined its expression in human placental villi and trophoblast cells. Immunohistochemical (IHC) staining (Fig. 3A) showed that specific SPARC expression could be detected in the cytotrophoblasts and syncytiotrophoblasts of human placental villi at 8 weeks of gestation. Negative control did not show any IHC staining (Fig. 3a). It was shown in Fig. 3A that specific brown colored staining for SPARC was recognized mainly in the cytoplasm but not in the nuclei of villous cytotrophoblasts and syncytiotrophoblasts cells (Fig. 3, b and c).

HTR8/SVneo cells, an invasive EVT cell line, were developed from an explant culture of human first-trimester placenta and shared many phenotypic similarities with the parental trophoblast cells without malignant phenotype (e.g. *in vitro* invasive abilities). We found that HTR8/SVneo cells had a much higher mRNA and protein expression of SPARC than JEG-3 cells, a cell line of choriocarcinoma origin (Fig. 3B). In order to investigate the role of SPARC in the trophoblast cells, we have designed and synthesize three SPARC siRNAs. As shown in Fig.3C, transfection with SPARC siRNA could markedly inhibit the expression of SPARC compared with that of control siRNA in HTR8/SVneo cells, and the protein levels of SPARC analyzed by Western blot correlated with the mRNA levels detected by RT-PCR.

### Downregulation of SPARC expression inhibits HTR8/SVneo cells invasion

**Figure 4:**
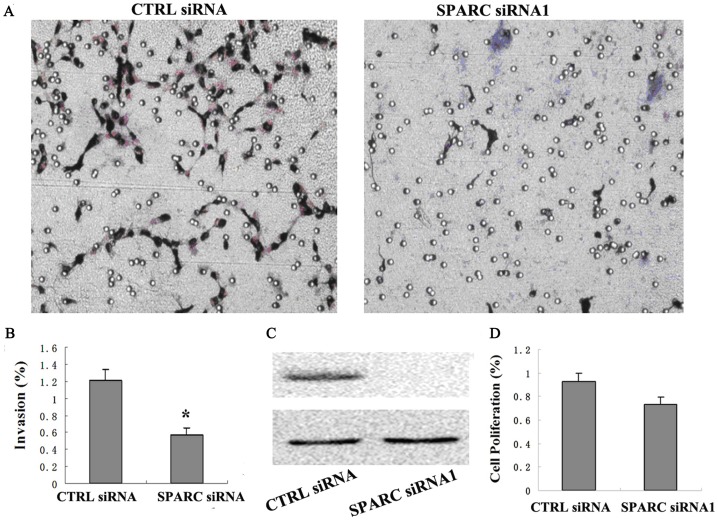
Silencing the expression of SPARC by RNAi resulted in decreased invasion capacity of HTR8/SVneo cells based on cell invasion assay. A. Representative images of HTR8/SVneo cells invaded through the filter were shown. B. Cell invasion were quantified by the invasion index as indicated as the ratio to the control siRNA. C. Confirmation of SPARC downregulation by RNAi was shown as Western blot results. GAPDH was used as a loading control. D. The effect of SPARC siRNA on proliferation of HTR8/SVneo cells by MTT assay. Each bar graph represents mean ± SD of samples from three independent experiments. Difference is considered to be significant at *p<0.05*.

The biological significance of SPARC expression on motility and invasion of HTR8/SVneo cells were evaluated using Boyden chamber assays. In the migration assay, HTR8/SVneo cells exhibited low serum-stimulated chemotaxis motility, and the loss of SPARC could slightly decrease the motility (data not shown). While the cells were subject to a Matrigel invasion assay, HTR8/SVneo cells transfected with SPARC siRNA had a significantly diminished invasive ability when compared with CTL siRNA (*P* < 0.05) (Fig. 4A and B). Western blot analysis indicated an efficient knockdown of SPARC expression in HTR8/SVneo cells after SPARC siRNA transfection (Fig. 4C). Moreover, a MTT assay was performed to examine the influence of siRNA 1 on the cell proliferation. Forty eight hours after transfection, there was no significant difference in cell viability between SPARC siRNA treated and CTL siRNA treated HTR8/SVneo cells (Fig. 4D).

### cDNA microarray analysis of the expression profile of SPARC downregulated HTR8/SVneo cells

**Figure 5:**
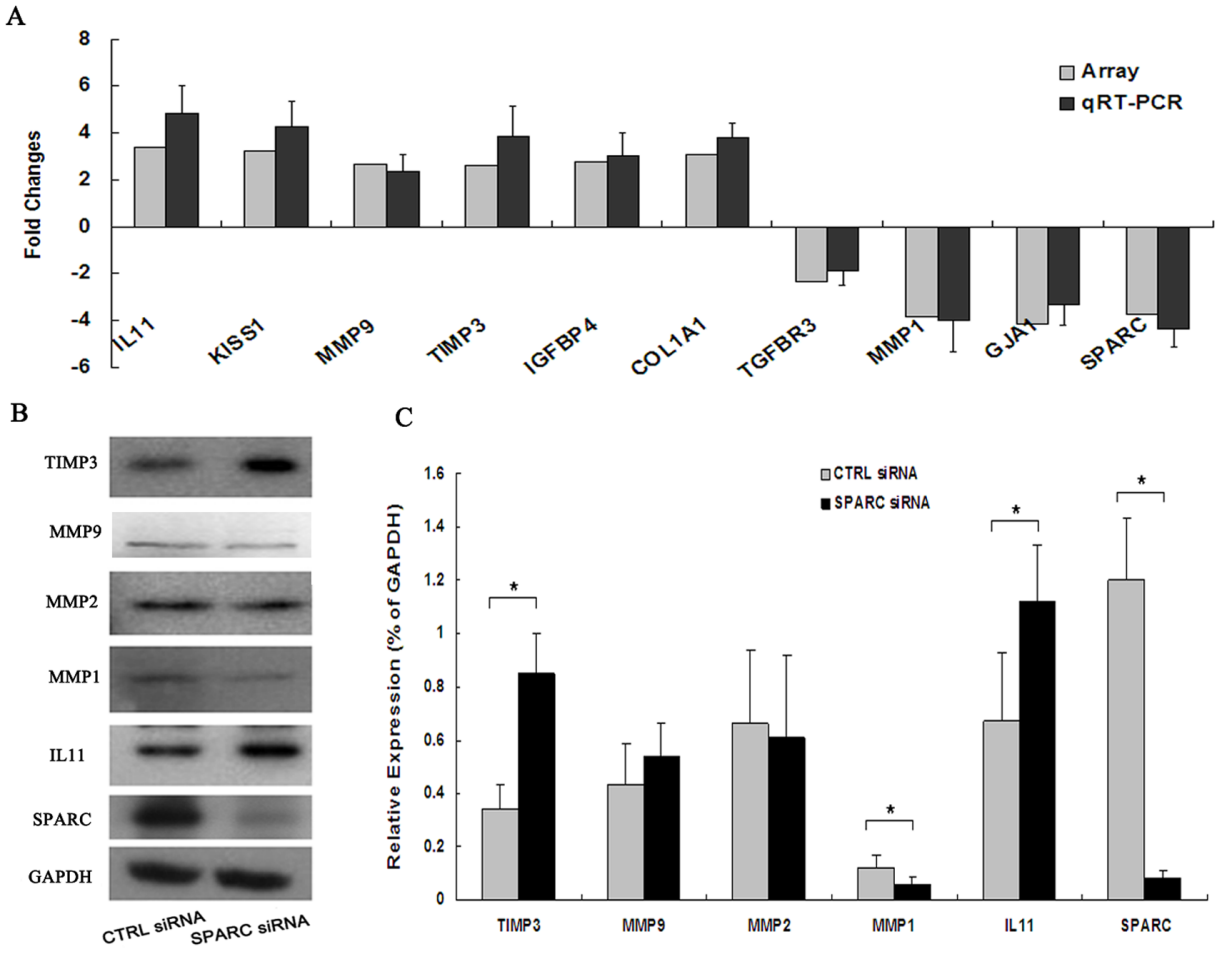
Alterations in the expression levels identified by microarray were confirmed using qRT-PCR and Western blot. A. qRT-PCR assay, mRNA levels were normalized with GADPH. B. Western blot analysis. C is a graphic representation of the relative protein expression levels in B. Data are mean values ± SD from three independent experiments performed in triplicate. Difference is considered to be significant at *p*<0.05.

To clarify the role of SPARC in trophoblast cell invasion, we performed cDNA microarray analysis using the human WG-6v3 Expression BeadChip containing more than 27,000 human genes. Microarray analysis revealed that when HTR8/SVneo cells were transfected with SPARC siRNA for 72 h, 282 genes were differentially expressed with a cut off set at 2-fold increase or decrease. Among these genes, 87 genes (31%) were upregulated, and 195 genes (69%) were downregulated. Table 2 and Table 3 list the top 25 genes in terms of either upregulation or downregulation, respectively. Gene function analysis revealed that gene clusters involved in ECM assembly and growth factor signaling ranked highly, involving genes such as collagen I (COLIA1), Interleukin 11 (IL11), insulin-like growth factor binding protein 4 (IGFBP4), gap junction protein alpha 1(GJA1), KISS1, matrix metalloprotease 9 (MMP9), et al. It is well-known that ECM components affect the behavior and function of trophoblastic cells by affecting matrix metalloproteases and their tissue inhibitors [25]. Growth factors and cytokines also play important roles in trophoblast cell invasion [7]. Therefore, the differences in the expression of these genes were further confirmed by real-time PCR. As shown in Fig. 5A, the results of qRT-PCR analysis were consistent with the expression profiles from the microarray hybridization data. These results indicated that our microarray data accurately reflect gene expression patterns. Western blot analysis (Fig. 5B) showed increased protein levels for TIMP3 and IL11, and a decreased protein level for MMP1 in SPARC knockdown HTR8/SVneo cells, which matched the expression patterns obtained from microarray analysis. However, although MMP9 mRNA level was observed to be markedly increased based on microarray analysis and real-time PCR, MMP9 protein level was not differentially expressed. A quantitative representation of the results was shown in Fig. 5C. It is evident from the results that SPARC downregulation in HTR8/SVneo cells led to increased gene expressions of COLIA1, IL11, KISS1, IGFBP4, MMP9 and TIMP3, but decreased expression of MMP1, TGFBR3 and GJA1.

**Table 2:** The top 25 genes upregulated in SPARC-knockdown HTR8/SVneo cells by microarray analysis.

	Gene symbol	Gene title	Fold-change ratio	Definition
	SPARC	Homo sapiens secreted protein,acidic, cysteine-rich (osteonectin)	0.27	The space external to the outermost structure of a cell
1	ACTG2	actin, gamma 2	4.79	All of the contents of a cell excluding the plasma membrane and nucleus, but including other subcellular structures
2	FAM55C	family with sequence similarity 55, member C	4.25	The space external to the outermost structure of a cell
3	ACLY	ATP citrate lyase	3.62	All of the contents of a cell excluding the plasma membrane and nucleus, but including other subcellular structures
4	C12orf49	chromosome 12 open reading frame 49	3.58	The space external to the outermost structure of a cell
5	TNFRSF10D	tumor necrosis factor receptor superfamily, member 10d	3.54	Penetrating at least one phospholipid bilayer of a plasma membrane
6	SYNGR3	synaptogyrin 3	3.48	Penetrating at least one phospholipid bilayer of a plasma membrane
7	IL11	interleukin 11	3.43	The space external to the outermost structure of a cell
8	LOC729310	hypothetical protein LOC729310	3.43	unknown
9	CRYAB	crystallin, alpha B	3.38	All of the contents of a cell excluding the plasma membrane and nucleus, but including other subcellular structures
10	H19	imprinted maternally expressed transcript	3.34	Unknown
11	KISS1	KiSS-1 metastasis-suppressor	3.23	The space external to the outermost structure of a cell
12	NEK7	never in mitosis gene a-related kinase 7	3.19	All of the contents of a cell excluding the plasma membrane and nucleus, but including other subcellular structures
13	SNORD13	small nuclelar RNA, C/D box 13	3.18	Unknown
14	COL1A1	collagen, type I	3.07	The space external to the outermost structure of a cell
15	LOC652815	similar to FK506-binding protein 9 precursor	3.02	unknown
16	APOE	apolipoprotein E	3.0	The space external to the outermost structure of a cell
17	ENC1	ectodermal-neural cortex (with BTB-like domain)	2.88	A membrane-bounded organelle of eukaryotic cells in which chromosomes are housed and replicated
18	RNF215	Homo sapiens ring finger protein 215	2.85	Penetrating at least one phospholipid bilayer of a plasma membrane
19	IGFBP4	insulin-like growth factor binding protein 4	2.76	The space external to the outermost structure of a cell
20	SLC22A18	solute carrier family 22, member 18	2.72	The membrane surrounding a cell that separates the cell from its external environment
21	LOC652388	similar to nodal modulator 2 isoform 2	2.71	Unknown
22	MMP9	matrix metallopeptidase 9	2.68	The space external to the outermost structure of a cell
23	USP14	ubiquitin specific peptidase 14	2.66	All of the contents of a cell excluding the plasma membrane and nucleus, but including other subcellular structures
24	LOC654135	similar to Acyl-protein thioesterase 2	2.65	Unknown
25	LOC653119	similar to block of proliferation 1	2.63	Unknown

**Table 3:** The top 25 gene downregulated in SPARC-knockdown HTR8/SVneo cells by microarray analysis.

	Gene symbol	Gene title	Fold-change ratio	Definition
	SPARC	Homo sapiens secreted protein, acidic, cysteine-rich (osteonectin)	0.27	The space external to the outermost structure of a cell
1	CPLX1	complexin 1	0.10	All of the contents of a cell excluding the plasma membrane and nucleus, but including other subcellular structures
2	MT1G	metallothionein 1G	0.12	Interacting selectively with copper (Cu) ions
3	SYP	Synaptophysin	0.15	Secretory organelles, some 50 nm in diameter, of presynaptic nerve terminals
4	LOC402644	similar to peptidylprolyl isomerase A isoform 1	0.16	unknown
5	TMEM198	transmembrane protein 198	0.19	Penetrating at least one phospholipid bilayer of a membrane
6	CHAC2	cation transport regulator homolog 2	0.18	The part of a cell or its extracellular environment in which a gene product is located
7	CGA	glycoprotein hormones, alpha polypeptide	0.19	The space external to the outermost structure of a cell
8	RPS26P10	ribosomal protein S26 pseudogene 10	0.19	Unknown
9	DNAJB4	Hsp40 homolog, subfamily B, member 4	0.19	The process of assisting in the covalent and noncovalent assembly of single chain polypeptides or multisubunit complexes into the correct tertiary structure
10	PLEKHA5	pleckstrin homology domain containing, family A member 5	0.19	The part of a cell or its extracellular environment in which a gene product is located
11	ECHDC1	enoyl Coenzyme A hydratase domain containing 1	0.21	The chemical reactions and pathways, including anabolism/catabolism, by which living organisms transform chemical substances
12	FOXG1	forkhead box G1	0.22	A membrane-bounded organelle of eukaryotic cells in which chromosomes are housed and replicated
13	GJA1	gap junction protein, alpha 1	0.24	The membrane surrounding a cell that separates the cell from its external environment
14	TNFSF18	tumor necrosis factor (ligand) superfamily, member 18	0.25	Penetrating at least one phospholipid bilayer of a membrane
15	TMEM167A	transmembrane protein 167A	0.26	Penetrating at least one phospholipid bilayer of a membrane.
16	MMP1	matrix metallopeptidase 1	0.26	The space external to the outermost structure of a cell
17	NR4A2	nuclear receptor subfamily 4, group A, member 2	0.26	A membrane-bounded organelle of eukaryotic cells in which chromosomes are housed and replicated
18	C12orf23	chromosome 12 open reading frame 23	0.26	Penetrating at least one phospholipid bilayer of a membrane
19	CYP51A1	cytochrome P450, family 51, subfamily A	0.26	The process of removal or addition of one or more electrons with/without the concomitant removal/ addition of a proton
20	TAF9	TATA box binding protein-associated factor	0.27	A membrane-bounded organelle of eukaryotic cells in which chromosomes are housed and replicated
21	DHRS2	dehydrogenase/reductase member 2 (DHRS2)	0.27	A membrane-bounded organelle of eukaryotic cells in which chromosomes are housed and replicated
22	FAM116A	family with sequence similarity 116, member A	0.28	unknown
23	NLK	nemo-like kinase	0.29	A membrane-bounded organelle of eukaryotic cells in which chromosomes are housed and replicated
24	MET	met proto-oncogene (hepatocyte growth factor receptor)	0.29	Penetrating at least one phospholipid bilayer of a membrane
25	MYO10	myosin X	0.30	Any of the various filamentous elements that form the internal framework of cells

## Discussion

It is known that SPARC is overexpressed in several invasive malignant tumors including meningioma, osteosarcoma and glioma. SPARC siRNA-transfected glioma cells failed to invade the surrounding normal brain tissue [26]. Since many similarities can be observed between invasive extravilloustrophoblast cells and cancer cells [3], [27], we hypothesized that SPARC might also be involved in the invasion process of EVT cells. Early in 1988, Wewer et al reported a substantial expression of SPARC in human decidua and carcinoma [28]. In the present study, immunohistochemistry analysis revealed a spatio-temporal expression of SPARC in the peri-implantation mouse uterus, indicating that SPARC may be an important reflection of the dynamics involved with tissue restruction during early pregnancy. The expression of SPARC was also detected in human placental villi (at 8 weeks) and trophoblast cell lines, and highly aggressive HTR8/SVneo cells showed more SPARC expression than JEG-3 cells. This observation supports that SPARC might have some potential roles in trophoblast invasion. Therefore, we examined whether or not the expression level of SPARC correlated with its invasive capacity in this trophoblast cell line. By using Boyden chamber assay, we found that knockdown of SPARC in HTR8/SVneo cells led to a significant decrease of the number of cells that had invaded through the matrix. In order to exclude the possibility that decrease in invasiveness potential of HTR8/SVneo cells by SPARC siRNA is a result of decrease in cell proliferation, we performed a MTT assay and the result indicated that SPARC siRNA did not suppress the proliferation of HTR8/SVneo cells. Furthermore, by Hoechst 33342 staining, SPARC downregulated HTR8/SVneo cells had no obvious apoptosis appeared when compared with control cells (result not shown). All together, our novel findings suggest that SPARC may functionally contribute to trophoblast cell invasion.

EVT invasion through the maternal decidua is critical to the formation of functional placenta. This process is tightly regulated by numerous growth and regulatory factors within the uterine endometrial microenvironment. Gene expression profile by cDNA microarrays has been recognized as a powerful approach to obtain a global view on gene expression and physiological processes in response to a particular stimulus. Therefore, we performed a cDNA microarray analysis using the human WG-6 v3 Expression BeadChip, and the results showed that, totally 844 genes were differentially expressed in SPARC knockdown HTR8/SVneo cells. Among the top 25 upregulated genes, 8 genes (32%) are ECM assembly molecules including APOE (apolipoprotein E), C12orf49 (chromosome 12 open reading frame 49), COLIA1, FAM55C (family with sequence similarity 55, member C), IGFBP4, IL11, KISS1, and MMP9. Among the top 25 downregulated genes, most of them are membrane-associated genes. We validated the expression of several SPARC target genes by real-time PCR, the result of which were identical to the profiles that were determined using microarray analysis. To gain further insight and independent support for the results, Western blot was performed to examine the protein levels of TIMP3, MMP9, MMP2, MMP1 and IL1. Although MMP9 mRNA level was observed to be markedly increased based on microarray analysis and real-time PCR, differential expression of MMP9 protein between SPARC downregulated HTR8/SVneo cells and control cells was not observed in Western blot assay. This difference was perhaps due to alternative transcriptional and translational steps and protein degradation.

Numerous studies suggest that SPARC functions as a regulator of tissue remodeling [29], [30], [31]. In fact, the phenotype of mice lacking SPARC validates the finding that SPARC is required for proper collagen matrix assembly and maturation [32], [33]. The SPARC target genes identified in this study are consistent with a potential ECM remodeling role in HTR8/SVneo cells. The trophoblast cells are confronted with various matrix protein and basement membranes, when penetrating the uterine wall. Collagen is the predominant ECM component, and collagen lysis is regulated by the balance between the activity of MMPs and TIMPs. In 2004, a global gene expression profile revealed an increase in mRNA levels of SPARC, collagens, MMPs and TIMPs in late radiation enteritis [34]. The SPARC and MMP9 are known to interact to regulate many stages of tumor progression. Forced expression of MMP9 rescues the loss of angiogenesis and abrogates metastasis of pancreatic tumors triggered by the absence of host SPARC [35]. Furthermore, SPARC overexpression induced a decreased expression of MMP9 and an increase of TIMP3 in medulloblastoma tumor [36]. Recently, it was reported that collagen deposits and mRNA expression levels were decreased in *SPARC* ^−/−^ mice when compared to *SPARC* ^+/+^ mice; in addition, MMP2 expression was increased in *SPARC* ^−/−^ mice [37]. However, the majority of these results could not reveal the molecular changes associated with trophoblast invasion. The present study demonstrates for the first time that SPARC downregulation led to significant increased gene expressions of MMP9, TIMP3 and COLIA1 in HTR8/SVneo cells, while MMP1 and GJA1 was decreased. In addition, the gene expression of chorionic gonadotropin (CG), a hormone indispensable for human pregnancy was found to be markedly reduced. Consistent with our finding, another group has demonstrated that CG increased migration and invasion of trophoblast cells [38].

Female mice with a null mutation in the gene encoding interleukin 11 receptor α (IL11Rα) are infertile due to disrupted decidualization, suggesting a critical role for IL11 and its target genes in the decidual response [39]. In 2004, White et al have found that uterine extracellular matrix components were altered during defective decidualization in *IL11Ra* ^−/−^ mice, and the gene expression of SPARC was upregulated determined by microarray analysis [40]. Consistent with this result, we observed a significant increase of IL11 in SPARC downregulated HTR8/SVneo cells, both at the mRNA and protein levels. In human placenta, insulin-like growth factors (IGFs) play important roles in syncytiotrophoblast steroidogenesis [41] and glucose and amino acid transport in the villi [42] and also in the invasion of EVT cells into the maternal decidua [43]. IGFBP4, the second most abundant IGFBP in the placental bed, is an inhibitor of IGF actions [44], and proteolysis of IGFBP4 enhances IGF bioavailability [45]. Early in 1994, Chandrasekhar et al reported that IL1 significantly inhibited the production and secretion of SPARC in rabbit articular chondrocytes. They also demonstrated that insulin-like growth factor IGF1 and transforming growth factor TGFβ1 could stimulate the synthesis of SPARC in these cells [46]. In our study, we found that SPARC depletion upregulated the expression of IGFBP4, but downregulated the expression of TGFBR3, indicating that the regulation of SPARC gene is complex and not completely understood. Expression of KISS1, a metastasis suppressor gene, was observed in trophoblast giant cells of the rat placenta in 2004 [47]. Further, KISS1 was identified to not only inhibit metastasis in various tumors, but also to repress trophoblast invasion via binding to the G protein-coupled receptor KISS1R [48]. In our study, for the first time, we demonstrated that KISS1 was regulated by SPARC in HTR8/SVneo cells.

Taken together, we demonstrated that SPARC plays an important role in implantation process, and suppression of SPARC expression inhibited the invasive capacity of trophoblast cells. cDNA microarray analysis reveals that hormones, ECM assembly molecules, growth factors and cytokines all are mediated by SPARC in EVT invasion. Further functional studies are required for clarifying the mechanism of SPARC action on trophoblast invasion. By elucidating the role of SPARC regulated genes in embryo implantation, new targets for the manipulation of human fertility may be identified in future.
